# Progress in Mesenchymal Stem Cell Therapy for Ischemic Stroke

**DOI:** 10.1155/2021/9923566

**Published:** 2021-06-15

**Authors:** Yinghan Guo, Yucong Peng, Hanhai Zeng, Gao Chen

**Affiliations:** Department of Neurosurgery, Second Affiliated Hospital, School of Medicine, Zhejiang University, Hangzhou, Zhejiang, China

## Abstract

Ischemic stroke (IS) is a serious cerebrovascular disease with high morbidity and disability worldwide. Despite the great efforts that have been made, the prognosis of patients with IS remains unsatisfactory. Notably, recent studies indicated that mesenchymal stem cell (MSCs) therapy is becoming a novel research hotspot with large potential in treating multiple human diseases including IS. The current article is aimed at reviewing the progress of MSC treatment on IS. The mechanism of MSCs in the treatment of IS involved with immune regulation, neuroprotection, angiogenesis, and neural circuit reconstruction. In addition, nutritional cytokines, mitochondria, and extracellular vesicles (EVs) may be the main mediators of the therapeutic effect of MSCs. Transplantation of MSCs-derived EVs (MSCs-EVs) affords a better neuroprotective against IS when compared with transplantation of MSCs alone. MSC therapy can prolong the treatment time window of ischemic stroke, and early administration within 7 days after stroke may be the best treatment opportunity. The deliver routine consists of intraventricular, intravascular, intranasal, and intraperitoneal. Furthermore, several methods such as hypoxic preconditioning and gene technology could increase the homing and survival ability of MSCs after transplantation. In addition, MSCs combined with some drugs or physical therapy measures also show better neurological improvement. These data supported the notion that MSC therapy might be a promising therapeutic strategy for IS. And the application of new technology will promote MSC therapy of IS.

## 1. Introduction

Stroke is the second leading cause of death in the world after ischemic heart disease [1]. Ischemic stroke (IS) accounts for 87% of all stroke patients, and its incidence rate is still rising [2]. Due to acute neurological deficits caused by focal cerebral ischemia, it has brought different degrees of disability burden to a large number of patients. Currently, there are few treatment options for ischemic stroke. Intravenous injection of tissue plasminogen activator (t-PA) can recanalize the blocked vessels. However, this treatment is limited by a short time window (≤ 4.5 hours) and the risk of secondary cerebral hemorrhage [3]. Mechanical thrombectomy (MT) can extend the treatment time window to 24 hours, but this kind of special operation can only be performed in a few qualified hospitals and needs to go through strict screening of indications and contraindications; only a few patients can accept MT treatment [4]. In addition, rehabilitation treatment can only bring limited functional improvement; there are still a large number of patients with permanent disabilities [5]. Therefore, it is imperative to develop a new treatment for ischemic stroke.

Stem cell therapy has been widely studied in different central nervous system diseases (such as autoimmune encephalomyelitis, spinal cord injury, and stroke) in recent 20 years [6–8]. There are many types of stem cells, including embryonic stem cells, neural stem cells, hematopoietic stem cells, and mesenchymal stem cells [9]. As early as 1970, Friedenstein et al. cultured fibroblast precursor from the cell suspension of guinea pig spleen and bone marrow, which is now called mesenchymal stem cell [10]. Later studies found that these cells have the potential of multidirectional differentiation. They can not only differentiate into mesoderm lineage cells such as osteoblasts, chondrocytes, adipocytes, and muscle cells [11–13] but also differentiate into endoderm and neuroectoderm cells, including endothelial cells [14], hepatocytes [15], neurons [16], and glial cells [17]. MSCs lack HLA-II molecules and rarely express costimulatory molecules, so they are nonimonogenic or hypoimmunogenic [18]. Due to its pluripotent differentiation and immune tolerance, MSCs have become one of the most promising candidate cells in stem cell therapy.

In 2006, the Mesenchymal and Tissue Stem Cell Committee of the International Society for Cellular Therapy (ISCT) proposed a set of minimum standards for the definition of bone marrow MSCs: First, MSCs must show the property of plastic adherence in standard culture conditions. Second, MSCs must express endothelial surface markers (CD73, CD90, and CD105) and be negative for hematopoietic markers (CD11b, CD14, CD19, CD34, CD45, CD79*α*, and HLA-DR). Third, MSCs must be capable of differentiating to osteoblasts, adipocytes, and chondroblasts in vitro [19].

In recent years, a large number of studies have proved that the application of MSCs can reduce the area of cerebral infarction after ischemia and promote the recovery of neural function. The therapeutic mechanism of MSCs in IS has not been fully understood, which may be related to its neuron replacement, neurogenesis, angiogenesis, and anti-inflammatory effects. The extracellular vesicles (EVs) produced by MSCs may also play an important role in this process. A large number of preclinical studies have proved its safety. However, there is no reliable and authoritative scheme for MSCs in the treatment of IS. The best choice of MSCs in source selection, treatment dose, treatment time window, administration method, and treatment strategy needs to be explored scientifically. This article will make a comprehensive review on the progress of MSCs in the treatment of IS.

## 2. Method

Review the literature and summarize the effects, strategies, related mechanisms, safety, and clinical application of MSCs in IS treatment through searching the PubMed database with key words: ((ischemic stroke[Title/Abstract]) OR (cerebral ischemia[Title/Abstract]) OR (cerebral infarction[Title/Abstract]) OR (middle cerebral artery occlusion[Title/Abstract]) OR (ischemic brain injury[Title/Abstract])) AND ((Mesenchymal Stem Cells[Title/Abstract]) OR (Bone Marrow Stromal Cells[Title/Abstract])). And a total of 296 results were presented over the past five years. We excluded 120 reviews, systematic review, comments, and irrelevant articles and finally got 176 research articles.

## 3. Mechanism of MSCs in the Treatment of IS

### 3.1. Immunomodulatory

Inflammation and immune response play an important role in the pathogenesis of stroke. After focal cerebral ischemia, reactive oxygen species, proinflammatory cytokines, and chemokines are released from damaged brain tissue, causing the activation of resident inflammatory cells such as microglia and astrocytes [20]. At the same time, a large number of inflammatory factors lead to the destruction of the blood-brain barrier, and blood-derived inflammatory cells enter the ischemic brain tissue, which expands the inflammatory response and activates the adaptive immune response [21, 22]. Although some studies have shown that inflammation after ischemia can promote the repair of brain tissue and the recovery of neural function in the chronic phase [23], more evidence supports the adverse effects of inflammatory response. The continuous infiltration of immune cells and the continuous expansion of the inflammatory response can cause neuronal necrosis, brain edema, and aggravate secondary brain injury [24, 25].

As mentioned above, inflammatory factors lead to the destruction of blood-brain barrier after ischemia, and then chemokines such as monocyte chemoattractant protein-1 (MCP-1) and stromal cell-derived factor-1 (SDF-1) can attract large-scale invasion of peripheral immune cells [21]. Researchers found that transplanted MSCs can maintain the integrity of blood-brain barrier and reduce the leakage of inflammatory cells in brain parenchyma by downregulating the expression of aquaporin-4 and reducing the release of neutrophil matrix metalloproteinase-9 (MMP-9) [26, 27]. In addition, MSCs can reduce the production of MCP-1 by secreting anti-inflammatory cytokine TGF-*β*, thus blocking the migration of CD68 + immune cells to the ischemic areas [28].

Studies have shown that the immunomodulatory effect of MSCs is related to the regulation of proinflammatory and anti-inflammatory cytokines. By coculturing MSCs with oxygen- and glucose-deprived (OGD) neurons, Huang et al. found that MSCs may play an anti-inflammatory role by secreting IL-6 and reducing the expression of proinflammatory cytokine TNF-*α* [29]. The potential signaling pathway may be related to the inhibition of NF-*κ*B activity by MSCs [30]. Similarly, in the animal model of IS, researchers also confirmed that MSCs can reduce the levels of proinflammatory cytokines TNF-*α* and IL-1*β* and reduce the infarct volume after focal cerebral ischemia [31]. Studies have shown that intra-arterial application of MSCs can inhibit the activation of acid sensing ion channels (ASICs) and then decrease the expression of the inflammasome, which leads to the inhibition of the activation of IL-1*β* [32]. Liu et al. reported that transplanted MSCs could alleviate nerve injury after focal cerebral ischemia by upregulating the expression of anti-inflammatory cytokine IL-10 and downregulating the expression of proinflammatory cytokine TNF-*α* [33]. A recent study showed that the above immunoregulation effect is achieved by MSCs enhancing Wnt/*β*-catenin signaling pathway, which is also related to MSCs mediated reduction of apoptotic cells after IS [34]. The cytokines involved in the immunoregulation of MSCs also include IL-23 and IL-17. In ischemic stroke, the inflammatory IL-23/IL-17 axis has been proved to be related to ischemia-reperfusion injury [35]. The experimental results of Ma et al. showed that MSCs injected via the caudal vein could reduce the infarct volume and promote the recovery of neurological function by downregulating the IL-23/IL-17 axis [36]. The above evidence suggests that MSCs can regulate the balance of proinflammatory and anti-inflammatory factors in the ischemic areas, and the same phenomenon is also observed in peripheral blood [37].

The activation of resident immune cells has a great impact on the inflammatory response after stroke. Previous in vitro experiments showed that MSCs could promote microglia to transform from a harmful neurotoxic phenotype, mainly releasing proinflammatory molecules to a beneficial neuroprotective phenotype producing anti-inflammatory molecules through CX3CL1 release [38]. Recently, Tobin et al. found that microglia in ischemic lesions of MSC-treated rats showed the same morphology of small cell bodies and a large number of branchings as that of inactive ones [39]. Similarly, the experimental results of Oh et al. showed that intravenous injection of MSCs could induce proinflammatory M1 microglia to differentiate into anti-inflammatory M2 microglia after IS and exert anti-inflammatory effects by increasing the expression of IL-1ra (an anti-inflammatory cytokine) [40]. Further studies show that the activation of cAMP-response element binding protein (CREB) induced by MSCs may be related to it [40]. In vivo and in vitro studies by McGuckin and colleagues have shown that MSCs can cause low expression of microglia activation markers (ED1 and Iba) and astrocyte proliferation markers (GFAP) [41]. It is suggested that the immunomodulatory effect of MSCs may be related to the inhibition of these two kinds of brain resident inflammatory cells, which may be related to a noncanonical JAK-STAT signaling of unphosphorylated STAT3 [41].

The proinflammatory effect of MSCs has also been reported. The experimental results of Li et al. showed that the release of inflammatory factors in the infarct lesions of MSC transplantation group increased on the second day after the establishment of the middle cerebral artery occlusion (MCAO) model but decreased on the seventh day [42]. Guan et al. reported that MSC transplantation increased the proportion of TNF-1*α* and IL-1*β* positive immune cells in the infarcted cortex of MCAO rats, which supported the immune-promoting effect of MSCs in the early stage of infarction [43]. However, MSC transplantation is still beneficial to the functional recovery of MCAO rats on the second day [42]. Therefore, more effort is required to further explore the exact mechanism of MSC-mediated immunoregulation in the pathological process following IS in the further.

### 3.2. Neuroprotection

After the occurrence of IS, the ischemic focus of brain tissue was formed, which was divided into the central ischemic core and the surrounding ischemic penumbra. Most cells died in the ischemic core, and the structure of the ischemic penumbra changed, but the neurons still survived [20]. With the prolongation of infarct time, hypoxia and hypoglycemia lead to the decrease of ATP production and cell death in ischemic penumbra neurons. Moreover, high levels of glutamate from the ischemic core can induce the production of apoptosis mediators such as nitric oxide and oxygen free radicals in ischemic penumbra and cause neuronal apoptosis [20]. Therefore, the protection of neurons in the ischemic penumbra is the key to the treatment of IS.

By inducing MCAO in rats, the researchers found that compared with the control group, the expression of antiapoptotic factor Bcl-2 protein in MSC group was significantly increased, and the expression of p53 protein was significantly decreased (the induction of p53 is related to neuronal apoptosis) [44]. In addition, the density of neurons around the ischemic area increased after MSC transplantation [44]. These evidences suggest that administration of MSCs can reduce neuronal apoptosis.

After transplantation, MSCs directly release or increase the release of endogenous neurotrophic factors such as brain-derived neurotrophic factor (BDNF) [45, 46], nerve growth factor (NGF) [47], glial cell line-derived neurotrophic factor (GDNF) [48], and basic fibroblast growth factor (bFGF) [49] to achieve neuroprotective effect. Chen et al. found that MSC treatment significantly increased bFGF in the ischemic border area of rats with middle cerebral artery occlusion, accompanied by a significant decrease of apoptotic cells in the ischemic border area [49]. In addition, the researchers found that transplantation of BDNF gene-modified MSCs could further increase the level of BDNF in the lesion area and further reduce neuronal apoptosis [45, 46]. The same enhancement effect was also observed in GDNF gene modified MSCs [48].

Studies have shown that fibronectin plays a neuroprotective role after IS. Fibronectin gene knockout mice show increased neuronal apoptosis and infarct size after transient focal cerebral ischemia [50]. The researchers found that six weeks after transplantation, transplanted MSCs still retained their fibronectin producing properties, suggesting that fibronectin may be involved in the neuroprotective effect induced by MSCs [51]. The imbalance of calcium ions in ischemic brain tissue after stroke can trigger the over activation of calcineurin (CaN) and cause neuronal apoptosis. Researchers found that MSC transplantation reduced the expression of CaN in the lesion, resulting in decreased neuronal apoptosis after IS [52].

In addition to apoptosis, MSCs can also play a neuroprotective role by alleviating parthanatos and necroptosis. By coculturing MSCs with OGD neurons, Kong et al. found that MSCs could protect neurons from parthanatos by reducing the nuclear translocation of apoptosis inducing factor (AIF) [53]. Moreover, the decrease of neuronal necrosis kinase RIP1 and RIP3 induced by MSCs was highly correlated with the decrease of neuronal necroptosis [53]. In addition, the experimental results of Nazarinia et al. showed that transplanted MSCs could reduce neuronal autophagy by increasing the expression of mTOR, thus playing a neuroprotective role after cerebral ischemia [54].

### 3.3. Angiogenesis

After IS, capillaries were destroyed, blood-brain barrier permeability increased, aggravating the inflammatory reaction, neuronal necrosis, and brain edema. Neovascularization after stroke helps to restore the blood and oxygen supply of the affected brain tissue, thus promoting nerve recovery, which may be a key factor in the survival of ischemic neurons [55]. The researchers found that patients with higher microvessel density at the edge of the ischemic area had a longer survival time, suggesting that poststroke angiogenesis plays an indispensable role in the prognosis of stroke patients [55].

Through three-dimensional analysis of the capillaries in the lesions, the researchers found that the number of new capillaries at the edge of the lesions in MCAO mice transplanted with MSCs increased significantly, which proved the role of MSCs in promoting angiogenesis [56, 57]. Studies have shown that neovascularization is mainly composed of endogenous endothelial cells but rarely differentiated from transplanted MSCs [57]. Moreover, there was no significant correlation between the microvessel density and the number of mesenchymal stem cells in the peri-infarct area [58]. Therefore, the replacement of vascular endothelial cells may not be the main mechanism of MSCs. The current view is that MSCs transplanted into the infarcted areas can promote angiogenesis by directly releasing or increasing endogenous nutrients such as vascular endothelial growth factor (VEGF) [29], angiopoietin-1 (Ang-1) [59], placental growth factor (PlGF) [60], and basic fibroblast growth factor (bFGF) 45-47 [61]. Both Ang-1 and VEGF have strong angiogenic effects, but their effects are not exactly the same. VEGF can promote the formation of immature vascular trunk, and Ang-1 participates in the maturation and stability of vessels [62, 63]. It has been reported that MSCs can reduce infarct size by releasing Ang-1 and VEGF to promote angiogenesis. Interleukin-1*β* may play an important role in this process [64]. Toyama et al. investigated the effects of Ang-1 gene-modified MCSs (Ang-MCS), VEGF gene-modified MCSs (VEGF-MCSs), and Ang-1 gene combined with VEGF gene-modified HMCS (Ang-VEGF-MCSs) on angiogenesis in infarcted area of MCAO rats and compared their therapeutic effects [56]. Both Ang-MCS group and Ang-VEGF-MCS group showed an increase in capillary volume and a decrease in infarct size, of which the Ang-VEGF-MCS group achieved the greatest benefit [56]. Surprisingly, transplantation of VEGF overexpressed MSCs can lead to increased infarct size and neurological deficits, which suggests that angiogenesis may require the coexpression of vascular endothelial growth factor and angiotensin-1 [56].

The signaling pathway of angiogenesis induced by transplanted MSCs remains to be explored. After induction of MCAO model, Guo et al. first confirmed the increase of neovascularization in the infarcted area of MSC-treated rats [65]. In addition, by Western blotting and double immunofluorescence staining, they found that the level of Notch 1 protein and Notch 1 positive microvessels in the lesion area increased significantly, suggesting that MSCs promote angiogenesis by activating the Notch signaling pathway in the endothelial cell of ischemic brain tissue after stroke [65]. Further study by Zhu et al. showed that the activation of Notch signal may be related to the secretion of VEGF-A by endothelial cells [66]. The administration of DAPT (a gamma secretase inhibitor, which can inhibit the activation of Notch signal) led to the decrease of vascular endothelial growth factor-A and the inhibition of angiogenesis after MSCs transplantation [66]. By coculturing the supernatant of MSC culture with human aortic endothelial cells, Hong et al. found that the former could inhibit hypoxia-induced endothelial cell apoptosis and promote angiogenesis [67]. This beneficial effect may be related to the activation of PI3K Akt signaling pathway, which may be one of the potential signaling pathways for MSCs to promote angiogenesis after IS [67].

### 3.4. Neural Circuit Reconstruction

Under suitable conditions, MSCs can differentiate into neurons and glial cells [68]. The original idea is that bone marrow mesenchymal stem cells can differentiate and replace damaged nerve cells after transplantation. However, although MSCs transplanted into the cortex around the infarcted area can express neuron-specific markers, the differentiated neurons are immature, with round shape and few fiber processes [51]. More importantly, they lack the voltage-gated ion channels needed to generate action potentials [69]. Therefore, the neural replacement mechanism may not be one of the mechanisms of MSCs in the treatment of IS.

After cerebral ischemia, endogenous neurogenesis occurs in the subventricular zone (SVZ) and the subgranular zone (SGZ) of the hippocampus. The newly formed neural progenitor cells can migrate to the infarcted area and further differentiate into neurons [70]. However, due to the unfavorable microenvironment full of inflammatory mediators and lack of nutrients after ischemia, most of these neural progenitor cells are facing the fate of rapid apoptosis, which limits the reconstruction of the neural network in the damaged area [70]. It is reported that MSCs can increase the number of neural progenitor cells and promote endogenous neurogenesis after IS [71, 72]. Through the electrophysiological recording of evoked field potentials, Song et al. found that the activity of neuronal circuits in the peri-infarct cortex of mice treated with MSCs was significantly increased [72]. Further studies have shown that MSCs can promote the migration and survival of neuroblasts to the ischemic penumbra and increase the number of neurons in the ischemic penumbra [72]. The increased expression of SDF-1 and polysialization enzyme induced by MSCs mediates the increased migration of neuroblasts to the injured site [73]. In addition, it is speculated that BDNF secreted by MSCs can promote the proliferation of neural stem cells in SVZ, increase the number of neural progenitor cells, and play a nutritional role in the process of proliferation, differentiation, and migration of neural progenitor cells, so as to prevent premature apoptosis [71, 74].

After IS, axonal sprouting and synaptic connection reconstruction of intact neurons promote the repair of neural function. However, the formation of glial scars in the ischemic area and the production of axon inhibitory proteins limit the reconstruction of the neural network [75, 76]. Liu et al. confirmed that the interhemispheric and intracortical axonal connections in the motor cortex around the infarction increased after stroke, and the application of MSCs significantly enhanced this effect [77]. Shen et al. demonstrated that MSC treatment significantly reduced the axonal loss and increased the expression of synaptophysin [78]. Further studies showed that MSCs could promote the reorganization of neural connections by reducing the thickness of the glial scars and the expression of Nogo-A (an inhibitor of axon growth) [78]. In addition, MSCs transplanted into the lesion may also promote axonal growth by downregulating the expression of neurocan (an axon elongation inhibitory molecule) and upregulating the expression of tPA in reactive astrocytes in the glial scars [75, 79, 80]. The above evidence indicates that MSC treatment can weaken the physical and chemical barrier effect of glial scars on axonal regeneration after infarction.

The transplanted MSCs may also promote axonal growth after cerebral ischemia by releasing nutrients. Song et al. showed that the expression of axon growth associated protein-43 (GAP-43) increased, and the expression of axon growth inhibitory proteins rock II and NG2 decreased in the cortex around the infarction in the MSC-treated mice, accompanied by the increase of axon density in this area. Further experiments showed that the increased expression of GAP-43 may be related to bFGF secreted by MSCs. In addition, in view of the role of BDNF in promoting and maintaining axonal branching, the effect of MSCs on axonal growth may also be related to the release of BDNF [81, 82].

After cerebral ischemia, the neural circuit reconstruction is affected by myelin reformation. It has been reported that transplanted MSCs can increase the number of oligodendrocyte progenitor cells in the peri-infarct area, corpus callosum, and SVZ [83, 84]. Recent reports by Tobin et al. also confirmed the above views. By measuring myelin basic protein in ipsilateral hemispheric tissue lysates of MCAO rats, the authors found that the total amount of myelin basic protein increased significantly after MSC treatment, suggesting that the role of MSCs in promoting neural circuit reconstruction also includes promoting myelin formation [39]. In conclusion, MSCs contribute to the reconstruction of neural circuits by inducing endogenous neurogenesis, promoting axonal budding and myelin regeneration, and specific signaling pathways remain to be investigated.

### 3.5. Mitochondrial Transfer

Transferring healthy mitochondria to damaged cells may be one of the mechanisms of MSCs in the treatment of ischemic stroke. Tunnel nanotubes (TNTs) are nanoscale tubular structures that connect adjacent cells. As a new intercellular communication mechanism, they can promote the exchange of components between adjacent cells [85]. By coculturing MSCs with human umbilical vein endothelial cells subjected to oxygen glucose deprivation and reoxygenation (OGD/RO), Liu et al. found that TNTs could be formed between MSCs and endothelial cells. Moreover, under the induction of OGD/RO, the functional mitochondria in MSCs transport to endothelial cells in a single direction, thus protecting endothelial cells from hypoxia injury [86]. In a paper published in 2019, the same author demonstrated that MSC transplantation after IS can also protect cerebral vascular endothelial cells through this intercellular connection. Their experimental results show that MSCs transplanted into the peri-infarct area can transfer their active mitochondria to the damaged microvascular endothelial cells, thus promoting angiogenesis, reducing infarct size, and improving neurological function [58]. Furthermore, the application of TNT inhibitors significantly reversed this effect, suggesting that TNTs play an important role in the mitochondrial transfer of this activity [58].

Besides vascular endothelial cells, MSCs can also transfer mitochondria to astrocytes and neurons damaged by oxidative stress, promoting their survival and proliferation [87, 88]. This beneficial effect also depends on the direct contact between cells, because the survival rate of neurons decreased after MSCs and neurons were separated by porous transmembrane [88]. In addition, Miro1, a mitochondrial Rho-GTPase 1, was upregulated in oxidative injured neurons and promoted the transfer of mitochondria from MSCs to neurons [87]. By coculturing Miro1 overexpressing MSCs with damaged neurons, the researchers found that more neurons survived, while Miro1 inhibited MSCs caused the opposite result [88]. Further in vivo experiments showed that Miro1 overexpressing MSCs could significantly improve neurological function compared with normal MSCs after transplanted into cerebral infarction animals [88]. In conclusion, the increased expression of Miro1 in neurons after IS can cause transplanted MSCs to transfer their healthy mitochondria to damaged neurons, thus increasing the metabolic activity or survival of neurons. The direct contact between MSCs and neurons and the establishment of TNT connections play an important role in this process.

### 3.6. EV Transfer

Mesenchymal stem cell-derived EVs (MSC-EVs) are spherical cytoplasmic components secreted by mesenchymal stem cells, which contain a large number of soluble bioactive components such as lipids, proteins, mRNAs, and microRNAs [89]. It can regulate the activity and function of target cells by combining with target cells and transferring the above cell components and genetic genes into target cells [89]. As a key messenger between MSCs and injured cells, MSC-EVs play an important role in the treatment of IS with MSCs.

By coculturing MSCs with OGD neurons and brain microvascular endothelial cells (BMEC), the researchers found that the former could reduce the apoptosis of damaged neurons and restore the tube formation of BMEC [90]. The addition of GW4869 (an inhibitor of EVs secret) can reverse this beneficial effect, which suggests that MSC-EVs may be the main mediator of the neuroprotective and angiogenic effects of MSCs [90]. Xin et al. injected rats with MSCs-EVs via the tail vein 24 hours after the induction of IS [91]. Compared with the control group, the density of axons and synaptophysin immunoreactive areas increased in the treatment group [91]. Immunofluorescence staining showed that the number of doublecortin (marker of neuroblasts) positive and von Willebrand factor (marker of endothelial cells) positive cells increased [91]. These evidences suggest that MSC-EVs can induce angiogenesis, neurogenesis, and neural circuit reconstruction after IS. Zhao et al. explored the anti-inflammatory effect of exosomes, the main components of MSC-EVs, in ischemic cerebral infarction. They confirmed that intravenous injection of MSC-derived exosomes 2 hours after IS resulted in a significant decrease in neurological severity score (NSS) and a significant improvement in motor function 7 days later [92]. In vitro, OGD microglia were cocultured with MSC-derived exosomes. It was found that the latter could inhibit the activation of M1 microglia, increase the number of M2 microglia, downregulate the levels of proinflammatory cytokines (TNF-*α*, IL-1*β*, and IL-12), and upregulate the levels of anti-inflammatory cytokines (TGF-*β* and IL-10) [92]. These data suggest that MSC-EVs are involved in immunomodulation, neuroprotection, angiogenesis, and neural circuit remodeling after transplantation of MSCs into the ischemic brain.

MSC-EVs may play a role by mediating the transfer of microRNA. First, Moon et al. demonstrated that 24 hours after MCAO induction, intravenous injection of MSC-EVs produced angiogenesis and neurogenesis, and this effect was positively correlated with the dose of MSC-EVs [93]. The contents of miR-184 and miR-210 in MSC-EVs were more abundant than those in fibro EVs [93]. Transfection of miR-184 and miR-210 into neural stem cells and human umbilical vein endothelial cells could increase the proliferation of these two cells, suggesting that MSC-EVs may induce the proliferation of vascular endothelial cells and neural stem cells after IS through miR-184 and miR-210 [93]. Secondly, MSC-EVs containing miR-29b-3p inhibitor could increase the apoptosis of oxygen-glucose-deprived neurons and decrease the angiogenesis of BMEC, while MSC-EVs overexpressing miR-29b-3p had the opposite effect [90]. This suggests that miR-29b-3p may mediate the neuroprotective and angiogenic effects of MSC-EVs. Mir-29b-3p may play a role by inhibiting PTEN and then activating Akt signaling pathway [90]. Furthermore, the experimental results of Geng et al. showed that MSC-EVs overexpressing miR-126 significantly increased the number of doublecortin positive and von Willebrand factor positive cells compared with normal exosomes [94], which suggests that miR-126 may be involved in EV-mediated angiogenesis and neurogenesis. Finally, MSC-EVs may play an indirect role in nerve repair after IS. In vitro experiments by Xin et al. showed that MSC-EVs overexpressing miR-133b could increase the secretion of exosomes by astrocytes, while the latter could significantly increase the number and length of axons [95].

**Figure 1:**
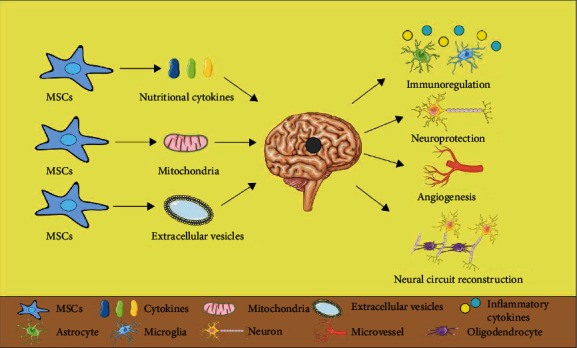
Mechanisms of MSCs in the treatment of IS. This figure contains elements available at Servier Medical Art repository, which is licensed under a Creative Commons Attribution 3.0 Unported License.

In conclusion, immunoregulation, neuroprotection, angiogenesis, and neural circuit reconstruction may be the main mechanisms of MSCs in the treatment of IS, while the secretion of nutritional cytokines, the transfer of mitochondria, and the transfer of extracellular vesicles may be the main ways of MSCs acting (Figure 1).

## 4. Selection of MSCs

### 4.1. Different Sources of MSCs

Bone marrow is the first tissue to isolate MSCs. However, the production of MSCs in bone marrow is low. The proliferation and differentiation potential of bone marrow-derived MSCs (BM-MSCs) decrease with age, and invasive bone marrow puncture is needed to obtain them [96], which makes bone marrow may not be the best source of MSCs. Besides, bone marrow, MSCs were also isolated from other tissues, including adipose tissue, placenta, umbilical cord, and dental pulp.

There are a large number of functional mesenchymal stem cells in adult adipose tissue. Adipose-derived MSCs (AD-MSCs) can be obtained by collagenase digestion of adipose tissue. Compared with BM-MCS, AD-MSCs are easier to obtain and cultivate enough autologous grafts [97]. Immunogenicity of human allogeneic AD-MSCs is lower than that of allogeneic BM-MCS [98], while autologous AD-MSCs show lower immunogenicity [99]. Due to its considerable clinical transformation potential, AD-MSCs are the most studied MSCs in IS besides BM-MSCs. Studies have shown that after the establishment of MCAO model, intravenous injection of AD-MSCs has the same curative effect as injection of BM-MSCs, and AD-MSC has even more advantages in reducing infarct size and improving neurological function [100]. Many other animal experiments have also proved that AD-MSC transplantation after IS has immunomodulatory effects [101–103], neuroprotective effects [102], angiogenesis effects [104], and neural circuit reconstruction effects [104]. It is worth noting that the experiment of Mangin et al. showed that intravenous injection of AD-MSCs could not improve the infarct size and neurological function after IS in diabetic or hypertensive mice [105]. Frutos et al. reported that intravenous transplantation of AD-MSCs can improve the function of hyperglycemic rats after cerebral infarction but has no effect on hypertensive rats [106, 107].

Dental pulp provides an accessible, noninvasive, high proliferation potential source of mesenchymal stem cells [108]. The extracted wisdom teeth are similar to the adipose tissue on the operating table. If they are not used for stem cell extraction or other purposes, these tissues will be discarded as clinical waste. Similar to BM-MSCs, the beneficial effect of dental pulp-derived MSCs (DP-MSCs) may be mediated by paracrine mechanisms rather than substitution [108, 109]. Song et al. compared the therapeutic effects of intravenous injection of human DP-MSCs and human BM-MSCs in MCAO model rats. There was no significant difference in the improvement of neurological function between the two groups, but the DP-MSC group showed smaller infarct volume [110]. In addition, the experimental results of Wu et al. showed that periodontal ligament stem cells (PDLSCs) were more effective than DP-MSCs in promoting the recovery of neural function after cerebral ischemia [111]. It is worth noting that a recent study reported that human DP-MSCs have the ability to produce action potentials after differentiation into neurons in vitro [112]. Whether they can be converted into functional neurons in animal models of IS remains to be explored.

Umbilical cord-derived mesenchymal stem cells (UC-MSCs) were extracted from umbilical cord perivascular tissue and Wharton's jelly (mucoid connective substance surrounding umbilical cord vessels). As the same as placenta, umbilical cord is easy to obtain as the waste after delivery, and there is no ethical problem. A number of studies have compared MSCs derived from the umbilical cord, dental pulp, bone marrow, and adipose tissue and found that MSCs derived from the umbilical cord have stronger proliferation activity [113, 114]. Studies have shown that UC-MSCs and placenta-derived MSCs (PL-MSCs) can alleviate neurological deficits after cerebral ischemia in rats, and their potential mechanisms are similar to those described above [96, 115–117]. It is worth noting that the data of Liao et al. showed that after transplantation of human UC-MSCs, more than 90% of the blood vessels around the cerebral ischemic area contained transplanted mesenchymal stem cells, which were integrated into the blood vessels and partially differentiated into endothelial cells [115]. The authors suggest that UC-MSCs can play the role of vascular remodeling by directly differentiating into vascular cells, which is not common in the experiment of using BM-MSCs.

Most of the BM-MSCs were extracted from long bone or iliac bone. Abiko et al. extracted BM-MSCs from rat skull and used them in MCAO model rats [118]. Their experimental results showed that compared with normal BM-MSCs, rats transplanted with skull-derived MSCs showed better neurological recovery, which may be related to the latter's ability to secrete more BDNF and VEGF [118, 119]. Interestingly, MSCs were also extracted from human turbinate. Lim et al. found that MSCs derived from human turbinate can promote neurogenesis after cerebral ischemia in rats, and the effect of improving neural function after IS is similar to that of AD-MSCs transplantation [120]. However, it is not easy to obtain MSCs from human skull and turbinate, and its clinical application potential is limited.

In addition to BM-MSCs, MSCs from adipose, dental pulp, umbilical cord, and placenta are the most promising types of MSCs for clinical treatment due to their easy availability and strong expansion ability. Future research also needs to clarify the consistency and difference of the mechanism of MSCs from different sources, and which kind of MSCs can obtain the maximum efficacy and the minimum adverse reactions in IS, so as to determine the most suitable source of MSCs for clinical application.

### 4.2. Autologous or Allogenic?

Although autologous MSCs are the safest, allogeneic MSCs have more advantages. First of all, autologous MSCs need a long time to culture and expand, which limits its application in the acute stage of IS, while allogeneic MSCs can be obtained and expanded from the freezer more quickly, thus avoiding the delay of time window. Second, patients with IS usually take antiplatelet or anticoagulant drugs, and the application of autologous MSCs may lead to secondary hemorrhage. Allogeneic MSCs from healthy donors have no such concerns. Third, age is a factor that affects the physiological characteristics of MSCs. Studies have shown that MSCs from elderly donors have decreased proliferation and differentiation ability, and the ability to secrete nutrients such as BDNF, VEGF, and insulin-like growth factor (IGF) is also affected [121–123]. Animal experiments have proved that transplantation of BM-MSCs from old people can improve the neurological function of rats after cerebral infarction and less than transplantation of MSCs from young people, and the effects of BM-MSCs from young people on anti-inflammation, angiogenesis, and secretion of nutritional factors are more significant [123]. However, IS patients are generally older, so allogeneic MSCs obtained from young healthy donors may be more effective.

## 5. Routes of Transplantation

### 5.1. Intraparenchymal Delivery

Intracerebral transplantation is effective in the treatment of experimental ischemic stroke [27, 124]. Direct injection of MSCs into the brain parenchyma can lead to the largest number of MSCs in the lesion area [27], with less side effects outside the brain, and may bring the best neurological improvement [124]. A meta-analysis of 46 articles showed that different routes of administration affected the therapeutic effect of MSCs in the treatment of IS [125]. Intracerebral administration was the best, followed by intra-arterial administration, and finally intravenous administration. However, neurosurgery is not acceptable to all patients [125]. Although the application of stereotactic technology can avoid craniotomy, it may still persecute the local brain parenchyma and blood-brain barrier, lead to additional neuronal damage and inflammatory reactions, and even cause complications such as hemorrhage and epilepsy. Therefore, whether the intracerebral delivery pathway is suitable for clinic application remains to be further studied and discussed.

### 5.2. Intraventricular/Intrathecal Delivery

Intraventricular or intrathecal injection of MSCs can spread to various parts of the central nervous system through cerebrospinal fluid. Lim et al. injected MSCs into the lumbar spinal cord sheath of rats with IS and found that compared with intravenous injection, MSCs injected intrathecally could effectively migrate to the infarcted area and could differentiate neurons and astrocytes, which promoted the improvement of motor function of rats with cerebral infarction [126]. A recent study reported that MSCs injected into the corpus callosum could migrate not only to the infarcted areas but also to the choroid plexus (CP) [127]. In vitro, the coculture of CP and MSCs showed that they could promote each other's proliferation, and this interaction may be related to neurogenesis [127]. Therefore, the authors suggest that MSC injected into the lateral ventricle containing CP is an appropriate way of transplantation. Although intraventricular or intrathecal administration is less likely to cause secondary injury than intraparenchymal administration, it is still more invasive than intravascular administration, and its inconvenient operation limits its clinical application.

### 5.3. Intravenous Delivery

The advantage of intravenous injection is that it avoids intracranial invasion, less trauma, and is simple and easy to operate. Many preclinical trials of MSCs in the treatment of IS have used intravenous injection and achieved good results, including the reduction of infarct volume and improvement of neurological function. Although different studies have reported that the therapeutic effect of the intravenous injection route on ischemic cerebral infarction may be the smallest, the benefits are still considerable [124, 125]. The limitation of intravenous injection is that it needs to reach the artery through the systemic venous circulation and then enter the brain through blood-brain barrier. The result of long-distance migration is that only a small part (4%) of MSCs injected intravenously can be located in ischemic brain tissue, and most of them stay in peripheral organs such as the lung, liver, spleen and kidney, and especially the lungs [47, 128]. Due to its large volume (18 *μ*m diameter), AD-MSCs have a higher pass clearance rate through the pulmonary circulation. The risk of intravascular injection lies in the possibility of pulmonary embolism. Cases of pulmonary embolism caused by intravascular injection of AD-MSCs have been reported in a journal, although it is not used for the treatment of IS [129].

### 5.4. Intra-arterial Delivery

Intra-arterial injection is another method of intravascular administration; most of the studies take internal carotid artery injection. Intra-arterial injection not only retains the advantages of minimally invasive intravascular administration but also can bypass the pulmonary circulation and improve the concentration of MSCs in the lesion. Li et al. injected 2 × 10^6^ MSCs into rats through the internal carotid artery and found that 21% of MSCs entered the brain and promoted the improvement of function after cerebral ischemia [130]. Zhang et al. compared the effects of intra-arterial, intravenous, and intracerebral transplantation in MCAO model rats and found that the intra-arterial pathway showed the greatest degree of neural function recovery [131]. The disadvantage of intra-arterial injection is that it may lead to the formation of intra-arterial emboli, decrease of cerebral blood flow, and cerebral microvascular embolism, and it is related to the dose of injected cells [132]. Clinical trials have proved the effectiveness and safety of intra-arterial injection of MSCs in patients with IS [133], so intra-arterial injection may be another suitable intravascular route.

### 5.5. Intraperitoneal Delivery

Intraperitoneal injection is a less used way of MSC administration. This way of administration can cause a large number of MSCs to accumulate in the abdominal cavity, while the number of MSCs migrating to other organs is very small [134]. An experiment in rats with hypoxic ischemic encephalopathy (HIE) model showed that the number of MSCs injected intraperitoneally homed to the ischemic area was less than that injected intravenously [135]. However, Barzegar et al. showed that intraperitoneal injection of MSCs showed a high survival rate [116]. These authors further demonstrated that intraperitoneal injection of MSCs into MCAO mice also showed an effective neuroprotective effect, which may be related to the significant recovery of cerebral blood flow after administration [116].

### 5.6. Intranasal Delivery

Intranasal route is a new way of stem cell transplantation. MSCs transplanted intranasally can bypass the blood-brain barrier at the nasal mucosa and then enter the brain through the olfactory sensory nerves or further transfer to other intracranial regions through cerebrospinal fluid [136]. Chau et al. reported that intranasal administration of MSCs reached the peri-infarct area 6 hours after delivery, and MSCs transplanted intranasally could reduce the infarct volume and promote the recovery of neurological function [137]. Zhao et al. showed that intranasal administration of MSCs can improve the permeability of blood-brain barrier and promote the recovery of neurological function after IS, which may be related to the promotion of revascularization in the infarcted area [138]. Researchers found that intranasal administration of MSCs in neonatal hypoxic-ischemic injury model mice can show the recovery of cognitive, sensory, and motor functions [139]. It is worth noting that intranasal administration of less MSCs can achieve the same effect as intracranial administration [139]. The advantages of intranasal injection lie in its noninvasive, simple operation, and repeated administration. However, compared with rodents, the human olfactory bulb is smaller. Whether intranasal administration of MSCs to IS patients can achieve the same effect as experimental animals remains to be proved by clinical trials.

In conclusion, in the current research on MSCs in the treatment of IS, intraparenchymal, intravenous, and intra-arterial administration are the three most widely used methods, and different administration methods have their own advantages and disadvantages (Table 1). Intravascular injection may be an appropriate route for MSC administration. Intranasal administration, as a new way of stem cell transplantation, has great potential in clinical application. Future studies need to evaluate and compare the safety and effectiveness of different ways of administration, so as to select the most suitable way of administration.

## 6. Timing of Transplantation

Different laboratories have studied the efficacy of MSCs transplanted at different time points after IS. Omori et al. divided the rats into two groups; one group was injected with 3 × 10^6^ MSCs at 6 hours after stroke induction, and the other group was injected with 1 × 10^6^ MSCs at 6 hours, 24 hours, and 48 hours, respectively [140]. The researchers found that the two groups achieved the same improvement in neurological function, suggesting that the time window of MSCs for the treatment of IS can be extended to 48 hours after cerebral infarction [140]. Hess et al. reported in a clinical trial that early administration of MSCs within 24-48 hours of the onset of symptoms of acute ischemic cerebral infarction may lead to a better one-year prognosis. Ishizaka et al. injected 1 × 10^6^ MSCs into the internal carotid artery of rats on days 1, 4, and 7 after MCAO induction (D1, D4, and D7). They found that the D1 group showed the earliest improvement of motor function, followed by the D4 group, but the D7 group did not recover. There was no significant difference in the degree of recovery between the D1 and the D4 groups [141]. This study expanded the treatment time window of MSCs to 4 days after IS and suggested that the early application of MSCs may get faster recovery. After intranasal administration of 1 × 10^6^ MSCs on the 3rd, 4th, 5th, and 6th day after IS, Chau et al. found that the motor function of mice was significantly improved on the 14th day, which suggested that the administration of MSCs in the delayed phase of IS was still beneficial [137]. In addition, it has been reported that MSCs can still significantly improve the neurological function of rats after 1 month of infarction, although it is used in a relatively large dose (3 × 10^6^) [142].

Due to the different ways of administration, dosage, and evaluation of neurological function in different studies, the best administration time cannot be obtained objectively. A meta-analysis involving 141 articles divided the administration time of these studies into four groups: 0-6 hours, 12-24 hours, 2-7 days, and > 7 days [143]. It was found that compared with the 12-24 hours group and > 7 days group, the score of comprehensive neurological function was significantly improved in the 2-7 days groups [143]. Moreover, there was no significant difference between 0-6 hours and 2-7 days, which suggests that 0-6 hours and 2-7 days after IS may be the best time for administration [143]. On one hand, in the early stage after IS, local brain tissue lacks oxygen and energy, and the inflammatory reaction is strong, which leads to the low survival rate of MSCs. Therefore, administration between 2-7 days after IS may increase the survival rate of MSCs, so that MSCs can play a greater role. On the other hand, the significant improvement of neural function induced by administration of MSCs at 0-6 hours may be related to the timely rescue of neurons in the ischemic penumbra, early intervention of inflammatory cells, and activation of the inflammatory cascade. In addition, the study also found that, compared with other groups, 0-6 hours administration caused the most significant improvement in sensorimotor outcomes [143]. Therefore, early administration within 7 days after stroke may be the best time for treatment.

Although the best time for MSCs to treat IS is still controversial, it is undeniable that MSCs expand the treatment time window of ischemic stroke, so that those patients who cannot receive tPA thrombolytic therapy for more than 4.5 hours can benefit from MSCs.

## 7. Doses of Transplantation

Studies have shown that intravenous transplantation of MSCs between 5 × 10^5^ and 3 × 10^6^ is effective in rodent stroke models [144]. In a preclinical study, MSCs were given at doses of 1 × 10^5^, 5 × 105, and 1 × 10^6^ 24 hours after stroke induction. It was found that compared with the control group, only rats in the 1 × 10^6^ group showed improved neurological function [40]. There are also studies using a relatively large dose (3 × 10^6^) of MSCS, which found that compared with the 1 × 10^6^ group, the infarct size of rats in the high dose group decreased by 20% and showed better neurological recovery. A phase I clinical trial has proved that intravenous infusion of 0.5 × 10^6^, 1.0 × 10^6^, and 1.5 × 10^6^ MSCs/kg allogeneic MSCs is safe and effective in patients with chronic ischemic stroke.

Is the greater the dose of MSCs, the greater the benefit? Lin et al. injected 1 × 10^6^ and 4 × 10^6^ human UC-MSCs intravenously 24 hours after MCAO in mice and found that a high dose of human UC-MSCs did not cause a more significant reduction of infarct size. The authors suggest that this may be due to the fact that most of MSCs remain in peripheral tissues after intravenous injection of a large doses of MSCs, so that the number of MSCs eventually reaching the central nervous system is not as much as expected [145]. A meta-analysis showed that the benefit of neurological function may not be directly proportional to the dose of MSCs but may be inverse *U*-shaped, as the benefit of behavioral function decreases at the highest dose of MSCs [125], which may be related to the disadvantages of intravascular administration. When MSCs are given a large dose via an artery or veins, it may cause microvascular obstruction or embolus formation and then decrease the perfusion of the brain or other organs. Therefore, it is necessary to grasp the relationship between effectiveness and safety to obtain the optimal dosage. More clinical and preclinical studies are needed to get the dose value corresponding to the apex of the inverse *U*-shape.

## 8. Therapeutic Strategy

Although MSCs have great potential in the treatment of ischemic stroke, due to the low homing rate, survival rate, and poor differentiation ability after transplantation, the effect of MSCs in the recovery of neurological function after cerebral infarction is still unsatisfactory, so researchers developed a variety of strategies to increase the efficacy of MSCs in ischemic infarction from different perspectives. The main strategies include pretreatment, gene transformation or overexpression, combination therapy, and MSC-EV transplantation.

### 8.1. Pretreatment of MSCs

Pretreatment is a strategy to change the culture environment of MSCs in different ways before transplantation to enhance their functional characteristics. Hypoxic culture is one of the most commonly used pretreatment methods for MSCs. Adapting to the hypoxic environment in advance may make MSCs play a better role in the face of barren environment in vivo. An appropriate hypoxia environment can increase the proliferation rate of MSCs and promote their differentiation into different mesenchymal cell lines in vitro [146]. By transplant of normoxic and hypoxic cultured MSCs into MCAO model animals, Hu et al. found that compared with the normoxic group, hypoxic preconditioning resulted in increased migration to the ischemic penumbra and improved survivability in adverse environments [147]. These benefits may be related to the increased expression of C-X-C chemokine receptor type 4 (CXCR4) in MSCs after hypoxia. As a ligand of SDF-1, the increased expression of CXCR4 promotes MSCs homing to the infarcted area [147]. Chen et al. showed that the enhanced migration and survival of MSCs after hypoxic preconditioning may be related to the inhibition of caspase-3 activation and the increased expression of hypoxia inducible factor-1*α* (HIF-1*α*) [148]. In addition, the expression of BDNF and VEGF in MSCs pretreated by hypoxia increased more significantly and promoted angiogenesis and nerve regeneration more significantly [148]. Moreover, the authors think that 8 hours is the best time for hypoxic preconditioning [148]. Kong et al. reported that MSCs cultured in a hypoxic environment expressed higher levels of CD200, which may be related to the decreased activation of microglia and the increased expression of anti-inflammatory cytokines such as IL-10 and TGF-*β* after MSC transplantation, suggesting that hypoxic preconditioning may enhance the immunomodulatory ability of MSCs after IS [117]. It should be noted that the transplantation of conditioned medium and exosomes derived from hypoxic preconditioning MSCs into MCAO model animals also showed a greater reduction of infarct size and improvement of neurological function [149, 150]. Moreover, hypoxic preconditioning can also improve the protective effect of conditioned medium derived from aged BM-MSCs on ischemic neurons, thus partially offsetting the adverse effect of age on the transplantation of autologous bone marrow stem cells [151].

In addition to culture in a hypoxic environment, there are many ways to pretreat MSCs. In vitro studies suggest that IL-1-treated MSCs can secrete more granulocyte colony stimulating factor (G-CSF) and reduce the secretion of inflammatory mediators in activated microglia [152]. The same authors injected medium derived from IL-1-treated MSCs into MCAO model animals and found that the conditioned medium could reduce the infarct volume by 30% 48 hours after stroke and improve the neurological function score [153]. The above evidence suggests that IL-1 pretreatment could induce MSCs to transform into anti-inflammatory and pronutritional phenotypes and play a beneficial role in cerebral infarction. Tobin et al. compared the efficacy of interferon-*γ* pretreated MSCs and normal MSCs in MCAO model animals and found that although there was no significant difference in functional improvement between the two, interferon-*γ* pretreated MSCs may have more advantages, which reflected in the fact that interferon-*γ* pretreated MSCs can induce activated microglia to secrete less proinflammatory cytokines and induce oligodendrocyte differentiation and myelination more effectively [39]. VX-765 is a selective caspase-1 inhibitor. Sun et al. found that transplantation of VX-765 pretreated MSCs resulted in more anti-inflammatory cytokines and less proinflammatory cytokines and apoptotic cells than nonpretreated MSCs. The enhanced anti-inflammatory and antiapoptotic effects of VX-765 may be related to the activation of autophagy by regulating AMPK/mTOR signaling pathway [154]. Different from the mechanism mentioned above, the authors believe that the increase of autophagy is beneficial to the treatment of IS by MSCs. Therefore, the role of autophagy in the treatment of is by MSCs remains to be further studied and explored.

MSCs-EV is one of the important mechanisms of MSCs in the treatment of IS. Cholesterol, as an important component of EVs, participates in the production, secretion, and functional regulation of EVs [155]. Barzegar et al. cultured human PL-MSCs with cholesterol lipid supplemented media and found that human PL-MSCs treated with cholesterol lipid could release more EVs, and the survival rate of vein transplantation was also significantly improved [116]. When 1 × 10^5^ human PL-MSCs treated with cholesterol lipid were intravenously injected into mice after MCAO induction for 1 hour, the researchers found that they could reduce the infarct size and restore neurological function, while the same low dose of common human PL-MSCs did not show any protective effect [116]. These evidences suggest that pretreatment of MSCs with cholesterol lipids can improve the efficacy of MSCs in the treatment of IS by enhancing the release and survival ability of EVs.

Three-dimensional (3D) aggregation is a new method of MSC culture. MSCs cultured in this way form a spheroid composed of 500-10000 tightly packed cells. Compared with 2D adherent MSCs, their migration ability and survival ability in hypoxia environment were enhanced, and the release of anti-inflammatory and nutritional factors was increased [156]. Yuan et al. verified the above characteristics of 3D aggregate-derived MSCs in MCAO model animals and showed smaller infarct size after transplantation of 3D aggregate-derived MSCs, suggesting that 3D aggregation is an effective pretreatment measure to enhance the efficacy of MSCs after IS [157]. Most stem cell studies use fetal bovine serum (FBS) to culture MSCs. Moon et al. compared the efficacy of MSCs cultured in fetal bone serum, normal health control serum, and stroke patient serum in MCAO model animals and found that rats transplanted with MSCs cultured in stroke patient serum showed more significant angiogenesis and neurogenesis, which is an inspiration for the way of autologous MSC transplantation [158].

### 8.2. Gene Transfection or Overexpression

It is a potential therapeutic strategy to enhance the therapeutic effect of MSCs in the treatment of IS by transfecting specific genes with viral vectors or plasmids to make MSCs overexpress certain molecules or proteins. After IS, the homing of transplanted MSCs is mainly mediated by the interaction between chemokine receptors on the surface of MSCs and high levels of chemokines in ischemic lesions. C-C motif chemokine ligand 2 (CCL2) is one of the most expressed chemokines in the ipsilateral cerebral hemisphere after IS. It mediates the transfer of a variety of cells to the brain by interacting with C-C motif receptor 2 (CCR2). Huang et al. transplanted CCR2 transgenic MSCs in MCAO model animal and found that this kind of CCR2 overexpression MSCs can more effectively migrate to ischemic lesions and mediate the protection of blood-brain barrier and the more significant improvement of neural function [159]. In addition, Lee et al. found that transplantation of CCL2 overexpressed MSCs resulted in a more significant increase in angiogenesis and neurogenesis and a more significant reduction in inflammatory response [160]. Moreover, the researchers also reported that overexpression of the neurogenic transcription factor neurogenin-1 can upregulate the expression of chemokine receptors CCR1, CCR2, and CXCR4 in MSCs, thus increasing the homing of MSCs to ischemic regions and promoting the further improvement of neural function [161]. The above evidence suggests that the more MSCs migrate to ischemic lesions, the more beneficial it will be. Promoting the homing of MSCs by genetic means is a potential therapeutic strategy.

Immunomodulation is an important mechanism of MSCs in the treatment of IS. As mentioned above, transplanted MSCs can regulate the inflammatory response after IS by upregulating the level of anti-inflammatory cytokines and downregulating the level of proinflammatory cytokines. Nakajima et al. reported that intravenous injection of IL-10 overexpressing MSCs resulted in a more significant reduction of infarct size and a more significant improvement of neurological function than normal MSCs [162]. Specifically, this kind of transgenic MSCs can lead to higher levels of anti-inflammatory cytokine IL-10 in lesions, which can inhibit the activation of microglia and the secretion of proinflammatory cytokines more effectively [162].

The repair of neural circuits is another important mechanism of MSCs in the treatment of IS. Noggin is an extracellular bone morphogenetic protein (BMP) antagonist, which promotes neurogenesis by inhibiting BMP signaling [163]. Chen et al. transplanted Noggin gene transfected MSCs intravenously 6 hours after induction of MCAO model and found that compared with the normal MSC group, this kind of MSCs could significantly increase noggin level in rat brain and more significant neurogenesis in ipsilateral SVZ [164]. Interestingly, Lu et al. transplanted MSCs cotransfected with BDNF gene and Noggin gene and found that these MSCs did not show additional antiapoptotic effects but showed additional anti-inflammatory effects [165]. Specifically, compared with MSCs transfected with BDNF gene or Noggin gene alone, CO transfected MSCs more effectively inhibited the activation of TLR4/MyD88 pathway and the expression of MMP-9 and reactive oxygen species (ROS) [165].

Since MSCs may play a therapeutic role mainly through paracrine, many researchers choose to overexpress some cytokines or nutritional factors to increase the therapeutic effect. Fibroblast growth factor 1 (FGF1), as a member of the paracrine FGF family, is abundant in neurons and can mediate neuroprotection [166, 167]. Ghazavi et al. investigated the effect of AD-MSCs transfected with FGF1 and found that compared with normal AD-MSCs, the former could increase the level of FGF1 in the ischemic lesions, reduce apoptotic cells, and infarct size more significantly [61]. In addition, Linares et al. found that FGF21 transfected MSCs have strong antiapoptotic ability in vitro in the face of oxidative stress and inflammatory environment, which suggests that FGF21 transfected MSCs may have stronger survival ability and neuroprotective ability in infarcted lesions [168]. LV et al. found that transplanted MSCs overexpressing HIF-1*α* had increased viability in ischemic lesions and showed a more significant reduction of infarct size and recovery of neurobehavior, which was related to the further decrease of proinflammatory cytokines and increase of neurotrophic factors [169]. HIF-1*α* is a protective regulatory factor produced by cells in the face of hypoxic environment. The therapeutic effect of HIF-1*α* overexpressing MSCs coincides with that of hypoxic preconditioning MSCs mentioned above. As mentioned in the mechanism section above, transplantation of VEGF expressing transgenic MSCs is not conducive to the treatment of IS. However, a recent study reported the opposite results. In this study, transplantation of VEGF expressing transgenic MSCs resulted in smaller infarct size, more significant angiogenesis, and neurological improvement [170]. The reasons for the two results are unknown, which may be related to different administration methods, administration timing, and measurement time points. Other studies transfected with different cytokines, such as BDNF, GDNF, PLGF, and hepatocyte growth factor (HGF), are shown in Table 2.

### 8.3. Combination Therapy

In recent years, the research focus of MSC treatment strategy is to enhance the efficacy of MSCs in the treatment of IS by combining with other drugs or treatment measures. Minocycline is a kind of tetracycline antibiotic. Due to its anti-inflammatory and antiapoptotic effects and good blood-brain barrier penetration, many studies have reported its beneficial effects in the central nervous system [171, 172]. Cho et al. showed that compared with transplanting MSCs alone, the combination therapy showed smaller infarct size and more significant improvement in neurological function, which may be related to minocycline enhancing the neurogenesis and angiogenesis of MSCs [173]. Simvastatin, as a class of HMG-CoA reductase inhibitors, was initially used to reduce cholesterol, and its application alone has also been proved to improve the prognosis after IS. Cui et al. found that Simvastatin can significantly increase the expression of the chemokine CXCR4 in MSCs, promote the homing of MSCs, and further promote angiogenesis and neural function recovery [174]. Combined therapy can also be achieved by intranasal administration. Shen et al. administered MSCs combined with IGF-1 into the nose and found that this strategy increased the ability of MSCs to promote angiogenesis and neurogenesis and further increased the cerebral blood flow in the ischemic area [175].

Because the repair of blood-brain barrier leads to the decrease of the passing rate of peripheral drugs, the drug treatment in the chronic stage of stroke often cannot achieve the desired effect. Although it has been reported that intravenous injection of MSCs at 1 month after stroke can improve motor function, this beneficial result may be attributed to the relatively large drug dose (3.0 × 10^6^) [142]. It has been reported that mannitol combined with temozolomide can inhibit the increase of blood-brain barrier permeability caused by endothelial tight junction proteins [176]. Choi et al. applied this strategy to MSCs in the treatment of IS and found that although MSCs were not detected in the brain parenchyma; these two drugs combined with MSCs could improve the behavior defect by increasing the brain parenchyma metastasis of MSC-derived microvesicles (a type of MSC-EVs), which was not observed in the MSCs only treatment group [177]. This combined strategy provides a new method for the treatment of chronic stroke.

Many traditional Chinese medicines have been proved to enhance the efficacy of MSCs in the treatment of IS. Radix Angelica Sinensis is a kind of Chinese herbal medicine with neuroprotective effects. Sodium ferulate (SF) and n-butylidenephthalide (BP) are the two main active components of Radix Angelica Sinensis. Study has shown that BP can enhance the interaction of SDF-1*α*/CXCR-4, promote MSCs to move to an ischemic focus, and promote MSCs to differentiate into astrocytes more effectively [178]. Zhang et al. injected SF, BP, and MSCs intravenously into MCAO rats and found that this combination therapy can further increase the levels of VEGF and BDNF in ischemic lesions and more effectively promote angiogenesis and neural function recovery [179]. Other research groups have also confirmed the enhancement effect of angelica extract on the efficacy of MSCs [180]. Tetramethylpyrazine (TMP), an active component extracted from Chinese herb Rhizoma Chuanxiong, has also been shown to increase the expression of CXCR4 in MSCs, thus promoting the homing of MSCs to infarcted brain tissue [181]. In the MCAO model animal, the combined application of TMP and MSCs further promoted the expression of VEGF and BDNF, resulting in a more significant improvement of neurological function score [182]. Other laboratory studies have shown that this enhancement effect may be related to the anti-inflammatory and neurogenesis effects of TMP on MSCs [183]. Other herbs with enhanced effects on MSCs in the treatment of IS include Salvia miltiorrhiza [184], Icariin [185], and Borneol [186].

In addition to drugs, some physical therapies combined with MSCs have also been proved to be beneficial. Morimoto implanted the electrical stimulator into the inner and outer sides of the cranial cavity of rats [187]. It was found that electrical stimulation could increase the movement of MSCs injected into the corpus callosum toward the ischemic focus, which was related to the increased level of SDF-1*α*. The rats in the combined group showed a smaller infarct size [187]. Electroacupuncture (EA) is a physical therapy that combines traditional acupuncture and electrical stimulation. Studies have shown that EA can increase the expression of BDNF and VEGF mRNA in a cerebral ischemia animal models and promote functional recovery [188]. Kim et al. treated mice with EA once a day from day 5 to day 16 after MCAO and found that EA combined with MSC transplantation could significantly improve the motor function of mice after cerebral infarction, which may be related to the promotion of neurotrophic factor secretion and neurogenesis [189]. Another report from the same laboratory showed that EA could increase the differentiation of TrkB gene transfected MSCs into mature neurons and increase the levels of BDNF and neurotrophin-4/5 more significantly [190]. Bi et al. placed the head on an ice bag for 3 hours immediately after MCAO induction and injected 1 × 10^6^ MSCs into the ventricle 24 hours later [191]. It was found that this mild hypothermia treatment increased the homing efficiency and angiogenesis ability of MSCs and significantly reduced the neurological function score [191]. In addition, MSC treatment combined with exercise or rehabilitation also showed a beneficial effect on the efficacy of MSCs [192, 193].

The rise of nanotechnology is of great help to regenerative medicine. Some new nanomaterials are used in the research of MSCs in the treatment of ischemic stroke. In vitro experiments showed that nitrogen-doped carbon nanocages (NCNCs) could enhance the inhibitory effect of MSCs on microglia activation [194]. Compared with transplanted MSCs alone, intravenous injection of MSCs combined with NCNCs in mice with cerebral infarction showed higher levels of IL-10, lower levels of TNF-*α*, and smaller infarct volume [194]. Nazarian et al. found that modafinil-coated gold nanoparticles (AuNPs) can promote the antiapoptotic ability of MSCs and further reduce the area of cerebral infarction, which is accompanied by a significant increase in the levels of BDNF and GDNF [195]. Zuo et al. reported that the combination of cerium oxide nanoparticles with human UC-MSCs could obtain the antioxidant effect of the former and enhance the anti-inflammatory effect of the latter. Specifically, the levels of ROS and inflammatory factors (TNF-*α*, IL-6, and IFN-*γ*) in the brain tissue of rats with cerebral infarction after transplantation of human UC-MSCs labeled with nanoceria were significantly decreased [196]. Yao et al. proposed a new nanoplatform to load MSCs. This method allows us to quantitatively detect cell migration by SPECT imaging after transplantation, and it can continuously release cobalt protoporphyrin IX to protect cells from oxidative stress, thus increasing the survival of MSCs in ischemic lesions [197]. The MCAO model mice transplanted with MSCs through this method showed better neurological recovery [197].

### 8.4. MSC-EV Transplantation

MSC-EVs may be one of the main mechanisms of MSCs in the treatment of IS. In recent years, many studies have used isolated MSC-EVs alone in the treatment of IS, showing that the curative effect is no worse than that of MSCs alone. Some studies even reported that injection of MSC-EVs alone showed more significant improvement in neurological function than MSC transplantation [93]. Therefore, transplantation of MSC-EVs alone seems to be a good alternative strategy, which has many advantages compared with MSCs. First of all, MSC-EVs injected intravenously can reach the infarct lesion more effectively [93]. Because of its smaller volume and lipid double-layer vesicle structure, MSC-EVs do not stay in peripheral organs like MSCs after intravenous injection and are easier to cross the blood-brain barrier. Secondly, the risk of vascular occlusion and microvascular thrombosis is reduced after transplantation, and MSC-EVs do not have the potential of tumor transformation because they cannot self-replicate. Third, MSC-EV has a robust structure and can be stored at -80° for a long time without loss of biological activity [198]. Finally, intravenous injection of MSC-EVs can reduce the peripheral immunosuppression (i.e., the decrease of B cells, NK cells, and T cells) after IS [95]. In addition, MSC-EVs can also be enhanced by genetic engineering. As mentioned above, MSC-EVs overexpressing certain miRNAs have stronger efficacy. Combination therapy also appears to be feasible, as AD-MSCs combined with AD-MSC derived exosomes intravenously administered 3 hours after IS resulted in smaller infarct size and better improvement in neurological function than either alone [199]. Therefore, MSC-EVs may be an effective alternative to MSCs, which has great potential in the treatment of ischemic stroke. At present, there are few clinical trials on MSC-EVs, and its efficacy and safety in stroke patients need to be further evaluated.

## 9. Clinical Trial

A large number of preclinical data have proved the feasibility of MSCs in the treatment of IS, and the clinical administration of stem cell therapy is also expected. A number of clinical trials have proved the effectiveness and safety of MSCs in the treatment of IS. The earliest clinical trial included only 30 subjects, five of whom received 1 × 10^8^ MSCs at 5-7 weeks after acute stroke. During the 1-year observation period, no adverse events were reported in these five patients. And the Basel index (BI) of these five patients was significantly improved, suggesting a certain improvement in neurological function [200]. Lee et al. enrolled 85 patients with severe IS. During the five-year follow-up, MSC treatment group had higher cumulative survival rate, more patients with low modified Rankin Scale (mRS) score (0-3), and no adverse reactions [201]. Additionally, a randomized controlled trial conducted by Jaillard et al. showed that although intravenous injection of autologous mesenchymal stem cells did not improve BI, mRS, and National Institutes of Health Stroke Scale (NIHSS) 2 years later, it promoted the improvement of motor function score [202].

In recent years, more and more different clinical trials have shown the possibility of diversified clinical transformation. A phase I/II clinical trial by Levy et al. demonstrated for the first time that a single intravenous injection of allogeneic BM-MSCs is safe and effective. None of the 15 serious adverse reactions during follow-up may be related to stem cell therapy. In addition, intravenous injection of 1.5 million cells/kg allogeneic MSCs in phase 2 showed significant improvement in BI score and NIHSS score [203]. Deng et al. conducted a phase II clinical trial to evaluate the safety and efficacy of intrathecal infusion of allogeneic BM-MSCs in the treatment of IS for the first time. 59 subjects received intrathecal infusion of allogeneic BM-MSCs four times a week (1 × 10^6^ cells/kg body weight), mainly to evaluate the mRS score and the occurrence of adverse events after 90 days of treatment [204]. The project is still in progress. Some literatures have also reported the clinical trials of modified MSCs in the treatment of IS. Steinberg et al. transplanted SB623 cells, BM-MSCs transfected with Notch-1 gene, into the brain of 18 patients with chronic stroke; results showed significant improvements in the European Stroke Scale (ESS) score, NIHSS score, Fugl-Meyer (F-M) total score, and F-M Exercise Scale score after 24 months of treatment [205]. There is a table of clinical trials of MSCs in the treatment of patients with IS (Table 3).

## 10. Discussion

In this study, we mainly focused on the mechanism, application parameters, and treatment strategies of MSCs in the treatment of ischemic stroke IS. On the one hand, the mechanism of MSCs in the treatment of IS was the focus of previous reviews [206, 207], but most of the previous articles in this area were not detailed enough. Here, we make a relatively comprehensive review and summary of the mechanism of action of MSCs and reviewed the research hotspots of the mechanism of action in recent years, namely, mitochondrial transfer and extracellular vesicles. We believe that this will provide a significant reference for the follow-up study of the mechanism of MSCs. On the other hand, application parameters and treatment strategies are the key to the clinical transformation of MSCs in the treatment of IS, but little attention of previous review was paid to these two aspects. This manuscript also makes a comprehensive review and summary of these two aspects, including the summary of the best application parameters of MSCs (i.e., the optimal dose and the optimal time window) and the display of the latest optimized treatment measures (i.e., pretreatment and combined treatment). We believe that this is of great significance to the development of follow-up clinical trials. In a word, we hope that both basic research and clinical trials can obtain useful information from this manuscript, so as to promote the progress of MSCs in the treatment of IS.

Immunomodulation, neuroprotection, angiogenesis, and neural circuit reconstruction are the main mechanisms of MSCs in the treatment of IS. Except for paracrine, mitochondrial transfer or extracellular vesicle transfer may also be the main pathway through which MSCs act, and MSC-EVs may be an effective alternative strategy for MSCs in the treatment of IS. MSC therapy extends the time window for treatment of ischemic stroke, and early administration within 7 days after stroke may be the best time for treatment. Intravascular injection of MSCs may be an appropriate way for clinical application, but we should pay attention to their adverse reactions. Intranasal administration is also a promising way of MSC transplantation. The optimal dose for treatment with MSCs is uncertain, but there is no positive linear correlation between dose and efficiency. There are a lot of researches on enhancing the therapeutic strategies of MSCs, but whether it is pretreatment or gene modification, combination therapy, or MSC-EVs, they are mainly based on the mechanism of MSCs in the treatment of IS. Clarifying the mechanism of action, molecular regulation, and signal pathways of MSCs will promote the discovery of more beneficial therapeutic strategies.

### 10.1. Limitation

Several limitations of the current review should not be ignored. First, we only reviewed the relevant literatures in PubMed database. Some articles not included in this database may be omitted, resulting in incomplete review. Secondly, in this study, there is no in-depth investigation and summary on MSC-EVs and micro-RNA which is the research hotpot of MSCs recently. Thirdly, the safety of MSCs in the treatment of IS, which is crucial to clinical transformation, has not been investigated in this study.

### 10.2. Future Directions

MSC therapy extends the time window for treatment of ischemic stroke, and early administration within 7 days after stroke may be the best time for treatment. Although the application of intravascular injection of MSCs may be an appropriate treatment for IS, more efforts might be required to determine the potential adverse reactions. And delivery MSCs through the intranasal route could also be a promising way of MSC transplantation. Furthermore, the optimal dose for treatment with MSCs needs to be investigated. Additionally, there are many different treatment strategies to optimize the efficacy of MSCs; researchers should carry out clinical trials in this area to achieve better clinical transformation in the future.

## 11. Conclusion

In summary, MSC transplantation provides hope for the treatment of IS. Further study of its mechanism and optimization of its treatment strategy will lay a solid foundation for the clinical transformation of MSC therapy.

## Figures and Tables

**Table 1:** Advantages and disadvantages of different MSC transplant approaches.

Routes	Advantages	Disadvantages	Reference
Intraparenchymal	Highest homing rate; low off-brain side effect	Highly invasiveness; additional brain tissue damage	[27, 124]
Intraventricular/intrathecal	Allow for migration to different parts of CNS; high homing rate	Invasiveness	[126]
Intravenous	Low invasiveness; easy operation	Stagnation in peripheral tissue; pulmonary embolism	[128, 129]
Intra-arterial	Low invasiveness; considerable homing rate	Microvascular occlusion	[130, 132]
Intraperitoneal	Low invasiveness; high survival rate	Low homing rate	[116, 134]
Intranasal	Noninvasiveness; easy operation; repeated administration	Lack of clinical trial evidence	[136–138]

**Table 2:** Gene transfection in the treatment of IS with MSCs.

Transfected gene	Transfected vector	Cell type	Dose	Transplantation route	Administration time	Outcome (compared with normal MSCs)	Reference
BDNF	Adenovirus	hBM-MSCs	5 × 10^5^	Intracerebral injection	24 hours after MCAO	Fewer apoptotic cells; smaller infarcted volume; improvement of neurological function	[45]
GDNF	Adenovirus	hBM-MSCs	1.0 × 10^7^	Intravenous injection	3 hours after MCAO	Smaller infarcted volume; higher function recovery	[48]
HGF	Herpes sim-plex virus type-1	BM-MSCs	1.0 × 10^6^	Intracerebral injection	2 or 24 hours after MCAO	Fewer apoptotic cells; smaller infarcted volume; higher function recovery	[208]
PIGF	Adenovirus	hBM-MSCs	1.0 × 10^7^	Intravenous injection	3 hours after MCAO	More angiogenesis; smaller infarcted volume; higher function recovery	[60]
FGF1	pCMV6-entry vector	AD-MSCs	2.0 × 10^6^	Intravenous injection	0.5 hour after MCAO	Fewer apoptotic cells; smaller infarcted volume; higher function recovery	[61]
Ang; VEGF; Ang+ VEGF	Adenovirus	hBM-MSCs	1.0 × 10^6^	Intravenous injection	6 hours after MCAO	Ang/Ang+ VEGF: more angiogenesis; smaller infarcted volume; higher function recovery. VEGF: infarct size increased; function deteriorated	[56]
VEGF	Adenovirus	BM-MSCs	1.0 × 10^6^	Intracerebral injection	24 hours after MCAO	More angiogenesis; smaller infarcted volume; higher function recovery	[170]
Hif-1*α*	Lentivirus	BM-MSCs	1.0 × 10^6^	Intracerebral injection	24 hours after MCAO	Lower level of proinflammatory cytokines; higher level of neurotrophins; smaller infarcted volume; higher function recovery	[169]
TSP4	Lentivirus	BM-MSCs	2.0 × 10^6^	Intravenous injection	3 hours after MCAO	Higher levels of Ang-1 and vWF; more angiogenesis; higher function recovery	[209]
IL-10	Adeno-associated virus	hBM-MSCs	1.0 × 10^6^	Intravenous injection	0 or 3 hours after MCAO	Lower level of proinflammatory cytokines and microglial activation; smaller infarcted volume; higher function recovery	[162]
CCR2	Lentivirus	BM-MSCs	2.0 × 10^6^	Intravenous injection	24 hours after MCAO	More homing; less BBB leakage; higher function recovery	[159]
CCL2	None	hUC-MSCs	1.0 × 10^6^	Intravenous injection	1 and 4 days after MCAO	More homing; more angiogenesis and neurogenesis; less neuroinflammation; smaller infarcted volume; higher function recovery	[160]
Ngn1	Retrovirus	hBM-MSCs	1.0 × 10^6^	Intra-arterial injection	2 hours after MCAO	More homing; fewer apoptotic cells; less neuroinflammation; higher function recovery	[161]
Noggin	Adenovirus	BM-MSCs	5.0 × 10^6^	Intravenous injection	6 hours after MCAO	More neurogenesis; smaller infarcted volume; higher function recovery	[164]

**Table 3:** Clinical trials of MSCs in the treatment of patients with IS.

Type of trial	Stroke type	Sample sizes	Cell type	Dose/single (*S*) or multiple (*M*)	Route	Time of adm. from stroke onset	Follow-up	Result	Reference
RCT	Acute IS	30	BM-MSCs/autologous	5 × 10^7^/*M*	IV	4-5 weeks 7-9 weeks	1 year	Significant improvement in BI. No significant difference in NIHSS and MRI scan	[200]
RCT	Acute IS	85	BM-MSCs/autologous	5 × 10^7^/*M*	IV	5 weeks 7 weeks	5 years	No significant side effects. Patients with mRS 0–3 significant increased	[201]
OL-PT	Chronic IS	12	BM-MSCs/autologous	0.6–1.6 × 10^8^/*S*	IV	36–133 days	1 year	No side effects. Decreasing of infarct volume by>20% at 1 week	[210]
OL-PT	Subacute IS	11	BM-MSCs/autologous	85 × 10^6^/*S*	IV	7–30 days	6 months	No side effects. Improvement in NIHSS, BI, and mRS	[211]
SB-CT	Acute IS	20	BM-MNCs/autologous	1.59 × 10^8^/*S*	IA	5–9 days	180 days	No side effects. No significant differences in neurological function	[212]
OL-PT	Chronic IS	36	BM-MSCs/allogeneic	1.5 × 10^6^/*S*	IV	>60 days	12 months	No side effects. Significant improvement in BI and NIHSS	[213]
OL-PT	Chronic IS	18	SB623 cells/allogeneic	2.5 × 10^6^/*S* 5.0 × 10^6^/*S* 10 × 10^6^/*S*	IC	>60 days	24 months	All experienced at least 1 treatment-emergent adverse event. Significant improvements in NIHSS F-M and ESS	[205]
RCT	Subacute IS	31	BM-MNCs/autologous	1.0 × 10^6^/*S* 3.0 × 10^6^/*S*	IV	<2 weeks	2 years	No significant improvements in NIHSS, BI, and mRs. Significant improvements in motor function	[202]
OL-PT	Chronic IS	12	BM-MNCs/autologous	Not provided	IV	3-24 months	4 years	No side effects. Significant improvements in mBI at 156 and 208 weeks	[214]

RCT: random control trial; OL-PT: open label prospective trial; SB-CT: simple blinded control trial; IV: intravenous; IA: intra-arterial; IC: intracerebral; adm: administration.

## Abbreviations

IS: Ischemic stroke
MSCs: Mesenchymal stem cells
EVs: Extracellular vesicles
MSCs-EVs: MSC-derived EVs
t-PA: Tissue plasminogen activator
MT: Mechanical thrombectomy
MCP-1: Monocyte chemoattractant protein-1
SDF-1: Stromal cell-derived factor-1
MMP-9: Matrix metalloproteinase-9
OGD: Oxygen and glucose deprived
ASICs: Acid sensing ion channels
CREB: cAMP-response element binding protein
MCAO: Middle cerebral artery occlusion
BDNF: Brain-derived neurotrophic factor
NGF: Nerve growth factor
GDNF: Glial cell line-derived neurotrophic factor
bFGF: Basic fibroblast growth factor
AIF: Apoptosis inducing factor
VEGF: Vascular endothelial growth factor
Ang-1: Angiopoietin-1
PlGF: Placental growth factor
bFGF: Basic fibroblast growth factor
Ang-MCS: Ang-1 gene modified MCSs
VEGF-MCSs: VEGF gene modified MCSs
Ang-VEGF-MCSs: Ang-1 gene combined with VEGF gene modified HMCS
SVZ: Subventricular zone
SGZ: Subgranular zone
GAP-43: Axon growth associated protein-43
TNTs: Tunnel nanotubes
OGD/RO: Oxygen glucose deprivation and reoxygenation
MSC-EVs: Mesenchymal stem cell-derived EVs
BMEC: Brain microvascular endothelial cell
BM-MSCs: Bone marrow-derived MSCs
AD-MSCs: Adipose-derived MSCs
DP-MSCs: Dental pulp-derived MSCs
PDLSCs: Periodontal ligament stem cells
UC-MSCs: Umbilical cord-derived mesenchymal stem cells
PL-MSCs: Placenta-derived MSCs
IGF: Insulin-like growth factor
HIE: Hypoxic-ischemic encephalopathy
CXCR4: C-X-C chemokine receptor type 4
HIF-1*α*: Hypoxia inducible factor-1*α*
3D: Three-dimensional
FBS: Fetal bovine serum
CCL2: C-C motif chemokine ligand 2
CCR2: C-C motif receptor 2
FGF1: Fibroblast growth factor 1
HGF: Hepatocyte growth factor
BMP: Bone morphogenetic protein
ROS: Reactive oxygen species
TSP4: Thrombospondin-4
SF: Sodium ferulate
BP: n-Butylidenephthalide
TMP: Tetramethylpyrazine
EA: Electroacupuncture
NCNCs: Nitrogen-doped carbon nanocages
AuNPs: Modafinil-coated gold nanoparticles
BI: Basel index
mRS: Modified Rankin Scale
NIHSS: National Institutes of Health Stroke Scale
ESS: European Stroke Scale
F-M: Fugl-Meyer total score.
